# Genetic Structure of Qiangic Populations Residing in the Western Sichuan Corridor

**DOI:** 10.1371/journal.pone.0103772

**Published:** 2014-08-04

**Authors:** Chuan-Chao Wang, Ling-Xiang Wang, Rukesh Shrestha, Manfei Zhang, Xiu-Yuan Huang, Kang Hu, Li Jin, Hui Li

**Affiliations:** 1 State Key Laboratory of Genetic Engineering and MOE Key Laboratory of Contemporary Anthropology, School of Life Sciences, Fudan University, Shanghai, China; 2 Key Laboratory of High Altitude Environment and Genes Related to Diseases of Tibet Autonomous Region, School of Medicine, Tibet University for Nationalities, Xianyang, Shaanxi, China; 3 CAS-MPG Partner Institute for Computational Biology, SIBS, CAS, Shanghai, China; IPATIMUP (Institute of Molecular Pathology and Immunology of the University of Porto), Portugal

## Abstract

The Qiangic languages in western Sichuan (WSC) are believed to be the oldest branch of the Sino-Tibetan linguistic family, and therefore, all Sino-Tibetan populations might have originated in WSC. However, very few genetic investigations have been done on Qiangic populations and no genetic evidences for the origin of Sino-Tibetan populations have been provided. By using the informative Y chromosome and mitochondrial DNA (mtDNA) markers, we analyzed the genetic structure of Qiangic populations. Our results revealed a predominantly Northern Asian-specific component in Qiangic populations, especially in maternal lineages. The Qiangic populations are an admixture of the northward migrations of East Asian initial settlers with Y chromosome haplogroup D (D1-M15 and the later originated D3a-P47) in the late Paleolithic age, and the southward Di-Qiang people with dominant haplogroup O3a2c1*-M134 and O3a2c1a-M117 in the Neolithic Age.

## Introduction

The Sino-Tibetan languages are a family of some 460 languages, including two subfamilies, namely Chinese and Tibeto-Burman. They are spoken by over a billion people all over East Asia and Southeast Asia, and second only to the Indo-European languages in terms of the population size of native speakers [1]. The linguistic connection between Chinese and Tibeto-Burman are well established. There are over 300 cognates between Old Chinese and Proto-Tibeto-Burman, grouping them into the same language family [1]. Based on lexical evidence and cladistic methods, Wang estimated that Chinese split away from Tibeto-Burman around 6 thousand years ago (kya) [2]. The Qiangic languages in western China were believed to be the oldest type of Sino-Tibetan languages, and have given birth to all other Sino-Tibetan languages [1]. Archaeological evidence [1], [3] also indicated that the ancestors of Sino-Tibetan populations lived around at least 6 kya in western China [1], [3].

Despite intense linguistic and archaeological researches, little has been known about how the Sino-Tibetan people dispersed from western China? During the past two decades, the characterization of genetic diversity has shed light on the history of Sino-Tibetan populations, especially the diversity defined by the maternal mtDNA and the paternal Y chromosome. In the maternal side, mtDNA evidence suggested a northern Asian origin of Tibetans, due to the high frequencies of northern Asian specific haplogroup A, D, G, and M8 [4]–[8]. However, that evidence has been contradicted by another work [9], which showed that the southern Tibeto-Burman populations exhibited sex-biased admixture with a stronger influence of northern immigrants on the male lineages and a more extensive contribution of southern natives to the female lineages. Likewise, the southern natives made a greater contribution to the maternal gene pool of southern Han Chinese [10].

Given that a correlation is emerging that suggests language change in an already-populated region may require a minimum proportion of immigrant males, while mtDNA types represent more ancient settlement [11], the Y chromosome characterization in the Sino-Tibetan populations may provide valuable insights into its origins. From the Y chromosome perspective, Su et al. found that almost all the modern Sino-Tibetan populations shared a common genetic signature, the high frequencies of O3-M122 lineages, including O3*-M122, O3a2c1*-M134, and O3a2b-M7. They postulated that the ancient Di-Qiang people (Proto-Sino-Tibetan speakers) with the dominant O3-lineages in the upper-middle Yellow River basin were the ancestors of present Sino-Tibetan populations [12]. However, they did not give a convincing explanation about the high frequency of Y chromosomal Alu insertion (YAP) in Tibetan populations. The YAP polymorphism was also enriched in Japan and Andaman islands, but basically absent in almost all the other East Asian populations [13]. Haplogroup D-M174 is one subhaplogroups of YAP+. Shi et al. proposed that D-M174 had a southern origin and then started its northward expansion about 60 kya. The current fragmented distribution of D-M174 was likely due to the later Neolithic expansion of Han culture carrying O3-lineages [14]. In addition, one of O3-M122 lineages in the study of Su et al. [12], haplogroup O3a2b-M7, was found out to be the characteristic lineage of Mon-Khmer and Hmong-Mien [15]. Haplogroup O3a1c-002611, which was included in the O3*-M122 haplogroup in the study of Su et al. [12], comprises almost 17% of Han Chinese [16]. However, haplogroup O3a1c was found at very low frequencies in Tibeto-Burman populations [17], suggesting that this lineage might not have participated in the establishment of the Tibeto-Burman populations. Recently, we have found that Qiang people have the highest Y chromosomal short tandem repeats (STRs) diversity among the Sino-Tibetan populations in the eastern Himalayas, indicating the Qiangic group to be the origin of the Sino-Tibetan expansion [18]. However, the highest genetic diversity of Qiang people might also be the result of repeated migrations from all directions.

**Figure 1:**
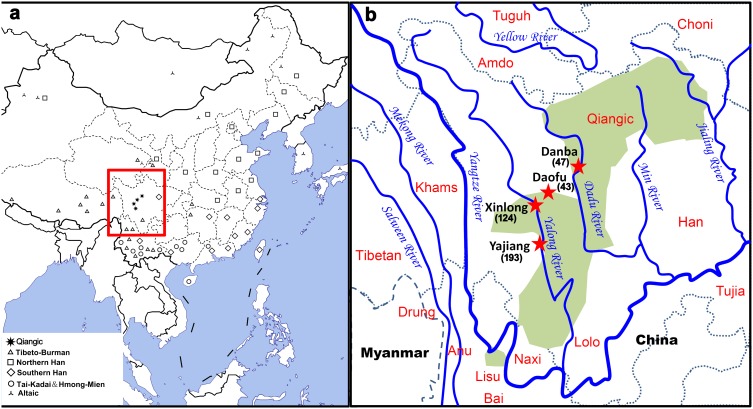
Geographic locations of Qiangic and other referenced East Asian populations in this study. (a). Geographic location of WSC and distributions of the East Asian populations used in data analysis; (b). Detailed geographic location of studied Qiangic speaking populations. The number of individual sampled in each population is enclosed in parentheses.

Y chromosome evidence indicates that Qiang people might be the origin source for the Sino-Tibetan populations [12], [18]. Qiang people refer to the populations speaking Qiangic languages, a group of the northeastern Tibeto-Burman branch, spoken mainly in Southwestern China (Figure 1), especially in western Sichuan (WSC). Qiangic has more than ten sub-branches, such as Horpa, Lavrung, Ersu and Zhaba [19]. The differentiation of the various Qiangic languages makes WSC a very important place for studying the origin of Sino-Tibetan. Furthermore, WSC is located between the upper-middle Yellow River basin and the eastern Himalayas, probably serving as a conduit for gene flow during the origin of the Sino-Tibetan populations. Here, we integrate Y chromosome and mtDNA diversity in Qiangic populations located in the WSC corridor to provide a broader framework for reconstructing the history of Sino-Tibetan.

## Materials and Methods

### Population samples

We collected blood samples of 407 healthy and unrelated individuals from four Qiaingic populations in western Sichuan province (Figure 1). Our study was approved by the Ethnic Committee of School of Life Sciences, Fudan University. All individuals were adequately informed and signed their informed content before their participation. The populations were labeled as follows: Horpa-Danba (DB), 47 Horpa individuals from Danba County of Sichuan; Horpa-Daofu (DF), 43 Horpa individuals from Bamei Town, Daofu County of Sichuan; Tibetan-Xinlong (XL), 124 Khams Tibetans from Xinlong County of Sichuan; Tibetan-Yajiang (YJ), 193 Khams Tibetans from Hekou Town, Yajiang County of Sichuan. Genomic DNA was extracted using DP-318 Kit (Tiangen Biotechnology, Beijing).

### Y chromosome markers

The samples were typed through seven panels of 100 SNPs as listed in the latest Y chromosome phylogenetic tree [16], [20].

Haplogroup O panel: M175, M119, P203, M110, M268, P31, M95, M176, M122, M324, M121, P201, M7, M134, M117, 002611, P164, L127 (rs17269396), and KL1 (rs17276338).

Corset Panel: M130, P256, M1, M231, M168, M174, M45, M89, M272, M258, M242, M207, M9, M96, P125, M304, M201 and M306.

Haplogroup C panel: P54, M105, M48, M208, M407, P33, M93, P39, P92, P53.1, M217, M38, M210, M356, P55, and M347.

Haplogroup D panel: P47, N1, P99, M15, M125, M55, M64.2, M116.1, M151, N2, and 022457.

Haplogroup N panel: M214, LLY22g, M128, M46/Tat, P63, P119, P105, P43, and M178.

Haplogroup R panel: M306, M173, M124, M420, SRY10831.2, M17, M64.1, M198, M343, V88, M458, M73, M434, P312, M269, and U106/M405.

Haplogroup Q panel: P36.2, M3, M120, MEH2, M378, N14/M265, M25, M143, M346, L53, and M323.

Those binary markers were hierarchically genotyped by SNaPshot (ABI SNaPshot Multiplex Kit) and fluorescent allele-specific PCR. PCR products were electrophoresed on a 3730xl Genetic Analyzer (Applied Biosystems, Carlsbad, CA).

Seventeen Y chromosomal STRs (DYS19, DYS389I, DYS389II, DYS390, DYS391, DYS392, DYS393, DYS385a, DYS385b, DYS438, DYS439, DYS437, DYS448, DYS456, DYS458, DYS635 and YGATAH4) were amplified using the AmpFlSTR Yfiler PCR Amplification kit (Applied Biosystems, Carlsbad, CA, USA). Amplified products were separated and detected using the ABI 3730xl Genetic Analyzer (Applied Biosystems, Carlsbad, CA, USA) according to the manufacturer’s recommended protocol. The data were analyzed using GeneMapper ID v3.2 (Applied Biosystems, Carlsbad, CA, USA). For use in the analyses, DYS389II was calculated by subtracting the DYS389I allele size.

### Mitochondrial DNA markers

The hypervariable segment I (HVS-I) of the control region was amplified by primers L15974 and H16488 [7]. Purified PCR products were sequenced using the BigDye terminator cycle sequencing kit and an ABI 3730XL genetic analyzer (Applied Biosystems, Carlsbad, CA, USA). A SNaPshot assay was used for typing SNPs in the coding regions to confirm haplogroup identity. This assay was designed as a multiplex panel including 21 coding region SNPs and one length variation marker [5]. Both the HVS-I motif and the coding region variations were used to infer haplogroups. In addition, three representative mtDNA (BM024, DBB005, and DBB006) have been completely sequenced using the method as described in our previous work [5]. The nomenclature of mtDNA follows van Oven and Kayser [21], with several latest new modifications (http://www.phylotree.org/). The mtDNA sequences have been deposited in Genbank with accession numbers KJ783504-KJ783899.

### Statistical analyses

Principal component analysis (PCA) was performed using SPSS 18.0 software (SPSS, Chicago, IL, USA). Networks of Y chromosomal STR data and the mtDNA HVS-I motifs were constructed by reduced median-joining method [22] using NETWORK v. 4.5.1.6 (Fluxus-engineering.com). Molecular diversity, population structure estimates and Y-STR genetic distances between populations were calculated using Arlequin v. 3.11 [23]. Classical frequency spectrum tests, such as Tajima’s D, Fu and Li’s D, D*, F and F*, were calculated using DnaSP5.0 to detect deviation from neutrality [24]–[27]. Coalescence times of mtDNA haplogroups of interest were calculated by ρ statistic method [28]–[29] using recently corrected calibrated mutation rate: 18,845 years per mutation in HVS-I (16090–16365) [30]. Reference population data on the Y chromosomes [14], [18], [31]–[44] and mtDNA [8]–[10], [45]–[69] were retrieved from the literature. Time estimations for main Y chromosomal lineages were made using 15 STRs (excluding DYS385a and DYS385b) in BATWING [70] under a model of exponential growth from an initially constant-sized population. The parameters used in estimation were following Xue et al [44]. Four sets of Y-STR mutation rates were applied in time estimations as Wei et al did [71]. These are a widely used evolutionary mutation rate (EMR) [72], two observed genealogical mutation rates (OMRB and OMRS) [73], [74], and a genealogical mutation rate adjusted for population variation using logistic model (lmMR) [73]. A total of 10^4^ samples of the program’s output representing 10^6^ MCMC cycles were taken after discarding the first 3×10^3^ samples as burn-in. The Time to the Most Recent Common Ancestor (TMRCA) is calculated using the product of the estimated population size N and the height of the tree T (in coalescent units) [70]. A generation time of 25 years was used to produce a time estimate in years. The geographic distributions of Y chromosome haplogroup D1 and D3a are presented by generation of contour maps using Surfer 8.0 Software (Golden Software).

## Results

### Y chromosome

#### Y chromosome haplogroup profile

**Figure 2:**
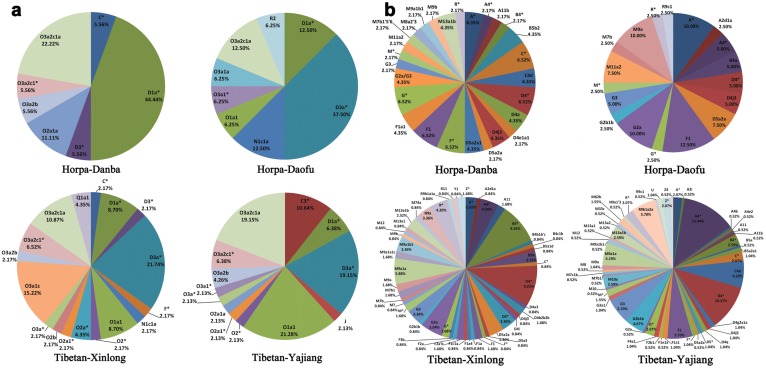
Y chromosome and mtDNA haplogroup frequencies of studied Qiangic populations. (a). Y chromosome haplogroup frequencies of the four Qiangic populations; (b). mtDNA haplogroup frequencies of the four Qiangic populations.

According to the nomenclature of Y Chromosome Consortium (YCC) [16], [20], 23 SNP haplogroups were determined from the 127 male individual samples (Figure 2a, Table S1, and Table S2). Haplogroup D1-M15 and its subhaplogroups, which are widely distributed across East Asia including most of the Tibeto-Burman, Tai-Kadai and Hmong-Mien speaking populations [4], [14], [75] (Figure S1 in Doc S1), are also prevalent in the four studied populations (44.44% and 12.50% in Horpa-Danba and Horpa-Daofu, respectively; 8.70% in Tibetan-Xinlong and 6.38% in Tibetan-Yajiang). Haplogroup D3a-P47 is almost exclusively distributed in Tibeto-Burman populations [4], [14], [75] (Figure S1 in Doc S1) and also found highly frequent in Horpa-Daofu, Tibetan-Xinlong and Tibetan-Yajiang, but absent in Horpa-Danba. Haplogroup O1a1-P203, which occurs at high frequencies in Tai-Kadai speaking people along the southeast coast of China and Taiwan aborigines [16], [75], is also observed at a high frequency in Yajiang (21.28%) and moderate frequencies in Daofu and Xinlong (6.25% and 8.70%, respectively), but absent in Danba. The major lineages in the Indo-China Peninsula, O2a1-M95 and its subhaplogroups, are also found at moderate or relatively low levels in the four studied populations. Haplogroup O3-M122 is the most common haplogroup in China and prevalent throughout East and Southeast Asia, comprising roughly 25–37% of the studied Qiangic populations. O3a1c-002611, O3a2c1-M134, and O3a2c1a-M117 are three main subclades of O3, each accounting for 12–17% of the Han Chinese [16], [75]. However, their frequencies vary a lot in Qiangic populations. O3a1c-002611 comprises 15.22% of Xinlong Tibetans, but absent in three other populations. O3a2c1*-M134 accounts for about 6% of the Horpa-Danba and Tibetans of Xinlong and Yajiang, but absent in Horpa-Daofu. Haplogroup O3a2c1a-M117, which exhibits high frequencies in other Tibeto-Burman populations, is also observed at high frequencies in Horpa-Danba and Tibetan-Yajiang (22.22% and 19.15%, respectively), and moderate frequencies in Horpa-Daofu and Tibetan-Xinlong (12.50% and 10.87%, respectively). Haplogroup C-M130 has a very wide distribution and might represent one of the earliest settlements in East Asia. Haplogroup C* (M130+, M105−, M38−, M217−, M347−, and M356−) has been found at low frequencies along the southern coast of mainland East Asia as well as throughout the islands of Southeast Asia [75], [76]. In spite of the wide distribution of C*, they all have similar STR haplotypes (DYS19, 15; DYS389I, 12; DYS389b, 16; DYS390, 21; DYS391, 10; DYS392, 11; DYS393). There are two C* individuals detected in this study, one in Horpa-Danba and the other in Tibetan-Xinlong. Those two individuals also have the same STR haplotype as mentioned above. Haplogroup C3-M217 is the most widespread subclade of C-M130, and reaches the highest frequencies among the populations of Northern East Aisa, especially in Mongolians [75]–[77]. Haplogroup C3-M217 has also been found in Tibetan-Yajiang at a frequency of 10.64%, but totally absent in other three populations. Haplogroup N-M231 has both a unique and widespread distribution throughout northern Eurasia and reaches highest frequency among most of the Uralic populations as well as some Altaic populations. Haplogroup N1c1a-M178 is the most common subclade of N-M231 and thought to be originated in China [75], [78]. N1c1a-M178 has also been detected in Horpa-Daofu and Tibetan-Xinlong at 12.50% and 2.17%, respectively. The 17-STR haplotype of N1c1a individuals in Horpa-Daofu is exactly the same with some Komi people in Russia [79], [80]. However, the haplotype of N1c1a individual in Xinlong shows more similarity with samples of its surrounding populations (unpublished data). It is particularly noteworthy that Central-South Asia related haplogroups J-M304 and R2-M124 [81] have also been detected at low frequencies in Qiangic populations.

#### PCA and STR genetic distance analysis

**Figure 3:**
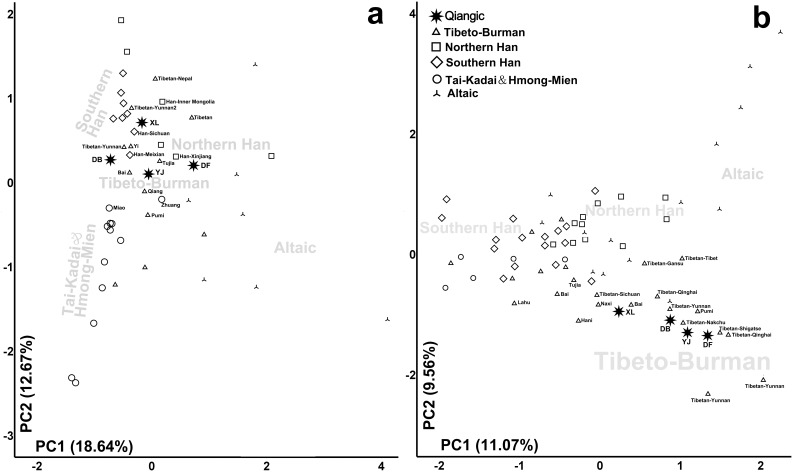
Phylogenetic relationship between Qiangic and reference populations analyzed by PCA with the frequencies of haplogroups. (a). PCA plot based on Y chromosome haplogroup frequencies of 51 populations; (b). PCA plot based on mtDNA haplogroups frequencies of 72 populations.

The paternal genetic relationships among Qiangic, Tibeto-Burman, and other East Asian populations were discerned with the aid of additional published Y chromosome datasets. We used a PCA based on the distribution of Y chromosome haplogroup frequencies of 51 populations to show the overall clustering pattern (Figure 3a, Table S3). Results of PCA are presented by the plots of the first two principal components (PCs), which together account for 31.31% of the Y chromosome variation in these populations. The first PC revealed a clear north-south geographic division between Altaic and Sino-Tibetan, Tai-Kadai & Hmong-Mien. Haplogroup C3-M217, G-M201, J-P209, and R-M207 were found to contribute most to the northern pole of Altaic. Haplogroup O-M175 contributed most to the southern pole. Sino-Tibetan, Tai-Kadai and Hmong-Mien populations showed different distributions of the second PC. Horpa-Danba, Horpa-Daofu, Tibetan-Xinlong, and Tibetan-Yajiang were clustered within Sino-Tibetan group, which reflected a clear linguistic clustering pattern. Haplogroups O3a1c-002611, O3a2c1*-M134, and O3a2c1a-M117 contributed most to the Sino-Tibetan pole. Contrastingly, haplogroups O3a2b*-M7 and O2a1-M95 were concentrated at the Tai-Kadai and Hmong-Mien pole. The four western Sichuan populations clustered tightly together with other Tibeto-Burman populations, such as Qiang, Tibetan-Yunnan, Yi, and Tujia, mostly due to high frequencies of haplogroup D3a-P47, O3a2c1a-M117, D1-M15, and O3a2c1*-M134. In the STR genetic distance based neighbor-joining tree, Horpa-Daofu, Tibetan-Yajiang, and Tibetan-Xinlong also clustered tightly with Tibeto-Burman populations. However, Horpa-Danba was close related to Han and Hmong-Mien populations (Figure S2 in Doc S1). As PCA was performed from frequencies of haplogroups and genetic distance was obtained from only 6 STR markers (Table S4), the results are suggestive but not conclusive.

#### Network analysis and time estimation

**Figure 4:**
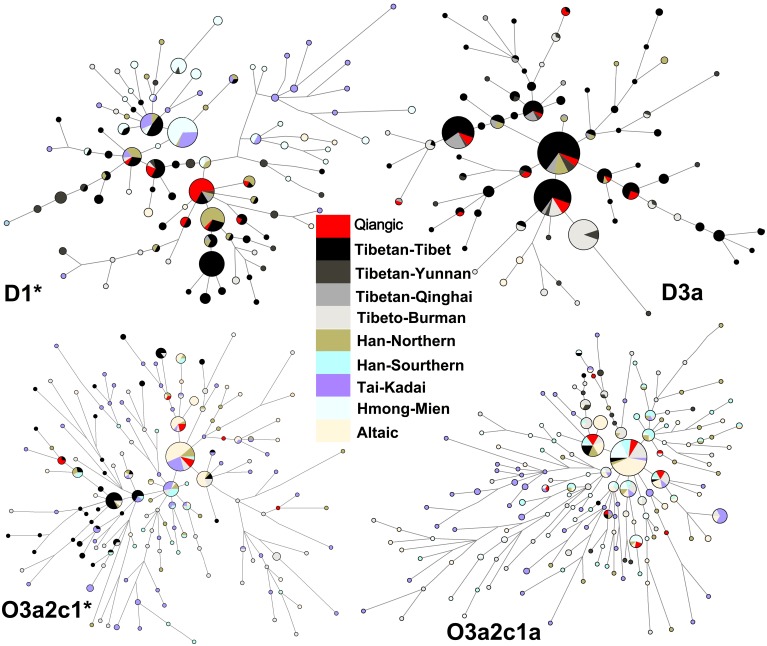
Reduced Median joining network of Y chromosome haplogroups. Reduced Median-joining network based on six Y-STR data (DYS19, DYS389I, DYS390, DYS391, DYS392, and DYS393) of haplogroup D1-M15, D3a-P47, O3a2c1*-M134, and O3a2c1a-M117.

To discern the detail relationship between the D3a-P47, O3a2c1a-M117, D1-M15, and O3a2c1*-M134 haplogroups in Tibeto-Burman and other related populations, a median-joining network was constructed based on Y-STR haplotypes of those haplogroups (Figure 4). A clear Sino-Tibetan vs. Tai-Kadai and Hmong-Mien divergence can be inferred from the network of D1-M15 though sporadic haplotype sharing exists. Furthermore, within the Sino-Tibetan populations, haplogroup D1-M15 contains distinct STR haplotypes between Qiangic populations, Northern Han, and Tibetan-Tibet, implying that D1-M15 experienced a serial of founder effects or strong bottlenecks and a secondary expansion in Sino-Tibetan populations. In the network of D3a-P47, the divergence between Qiang and Tibetan with other Tibeto-Burman populations has been observed. Other Tibeto-Burman populations only have a subset of the Qiang and Tibetan haplotypes. The star-like network of D3a-P47 also suggests population expansion in Tibetans. The network of O3a2c1*-M134 shows a clear divergence between Tibetan and northern populations (Northern Han and Altaic). Southern Han and Tai-Kadai samples constitute the center of the network and act as a bridge connected Tibetan and northern populations, which supports the southern origin and northern expansion of O3a2c1*-M134. Most of the Qiangic samples belonging to haplogroup O3a2c1*-M134 share haplotypes with northern populations, indicating a recent gene flow from northern populations to Qiangic populations. A population expansion has also been observed in the star-like network of haplogroup O3a2c1a-M117. o However, the haplotypes of O3a2c1a-M117 are extensively shared among all the East Asia populations.

We then estimated the coalescence and expansion time of Y chromosome lineages in Qiangic populations (Table 1). The ages estimated using evolutionary rate are about two or three times higher than using genealogical rates. As the times using genealogical rates fit well with sequence-based estimates in Y chromosome lineage dating [82], we present results from the genealogical calculations in the following section. Haplogroup D can trace back to late Palaeolithic period, while other subhaplogroups coalescence more likely in Neolithic Time. The lineage expansion times all fall into Neolithic Time ranging from 4.2 to 7.5 kya.

**Table 1:** Estimates of the coalescence time of selected Y chromosome haplogroups within Qiangic populations using Batwing.

Mutation rate			D	D1a	D3	D3a	O1a1	O3a1c	O3a2c1*	O3a2c1a
OMRB	TMRCA (years BP)	mean	16828	9552	7284	5393	8054	11364	12087	7167
		median	13807	8070	5625	4113	6683	8198	8892	5491
		SD	3804	1836	2161	1633	1698	4342	4385	2158
	Expansion (years BP)	mean	7928	6862	6295	5820	6609	10788	10169	7117
		median	5548	5103	4589	4261	4975	7490	7050	5205
		SD	3031	2261	2345	2139	2170	4896	4628	2666
OMRS	TMRCA (years BP)	mean	14657	7699	6499	4432	7009	9408	9749	6097
		median	12065	6509	5069	3425	5837	6799	7158	4717
		SD	3268	1459	1865	1273	1432	3579	3580	1768
	Expansion (years BP)	mean	7302	5827	5728	5213	5789	9448	8974	6412
		median	5165	4373	4209	3864	4367	6560	6242	4738
		SD	2732	1874	2080	1843	1870	4325	4081	2336
lmMR	TMRCA (years BP)	mean	16339	9493	7019	5461	7592	11121	11724	7163
		median	13368	7997	5403	4162	6280	8002	8576	5441
		SD	3788	1857	2081	1663	1633	4288	4352	2217
	Expansion (years BP)	mean	7828	6898	6326	5899	6638	10734	10098	7180
		median	5509	5141	4627	4319	5017	7437	6973	5217
		SD	3005	2285	2326	2171	2184	4892	4656	2734
EMR	TMRCA (years BP)	mean	61245	37303	23989	19642	31365	47036	42244	26666
		median	50014	30316	18544	15078	24777	34581	31205	20191
		SD	13963	8828	6950	5825	8457	16827	14929	8404
	Expansion (years BP)	mean	17964	17671	16601	15520	19721	34503	30242	19886
		median	11959	11379	12060	11355	13753	24220	21250	14185
		SD	7870	8224	6352	5817	8648	15101	13198	8162

Abbreviations: BP, before present; SD, standard deviation; Expansion: population expansion time; TMRCA: Time to the Most Recent Common Ancestor; EMR: evolutionary mutation rate [72]; OMRB and OMRS: two observed genealogical mutation rates [73], [74]; lmMR: genealogical mutation rate adjusted for population variation using logistic model [73].

*is a part of the haplogroup name.

### MtDNA

#### MtDNA haplogroup profiles, Population summary statistics, and PCA analysis

MtDNA HVS-I sequences of 396 individuals from the four studied Qiangic populations have been successfully typed. A total of 214 different haplotypes were defined by 134 polymorphic sites in the HVS-I dataset. The haplotype diversity of those Qiangic groups ranged from 0.978 to 0.994, with the lowest haplotype diversity observed in Horpa-Daofu (0.978) and the highest in Horpa-Danba (0.994). The mean number of pairwise differences (MNPD) and nucleotide diversity (ND) show a similar pattern with the haplotype diversity, as the highest diversity was observed in Horpa-Danba and the lowest in Horpa-Daofu. However, Tibetan-Yajiang has a higher diversity in haplotype but lower diversity in MNPD and ND than Tibetan-Xinlong. Measures of population growth (Tajima’s D, Fu’s Fs, Fu and Li’s D*, and Fu and Li’s F*) all gave the negative values for each population, but Tajima’s D, Fu & Li’s D* and F* were not statistically significant in Horpa-Daofu (Table 2). The not significant growth factor values and the lowest diversities of Horpa-Daofu might be the result of small sample sizes and/or genetic drift.

**Table 2:** Molecular diversity indices and growth summary statistics for Qiangic populations.

	N	h	S	Diversity Indices			Growth Statistics							
				HaplotypeDiversity(SD)	MNPD(SD)	NucleotideDiversity(SD)	Tajima’s D	P	Fu’s Fs	P	Fu and Li’s D*	P	Fu andLi’s F*	P
DB	43	39	61	0.994±0.00005	6.468±3.121	0.0140±0.0075	−1.927	0.007	−25.174	0.0000	−2.199	>0.05	−2.515	<0.05
DF	43	31	38	0.978±0.00012	5.126±2.534	0.0111±0.0061	−1.443	0.063	−25.500	0.0000	−2.187	>0.05	−2.312	>0.05
XL	122	88	87	0.993±0.00001	5.698±2.749	0.0123±0.0066	−2.081	0.001	−25.187	0.0000	−3.437	<0.02	−3.434	<0.02
YJ	188	97	91	0.980±0.00001	6.027±2.884	0.0130±0.0069	−1.906	0.007	−24.916	0.0000	−2.729	<0.05	−2.863	<0.05
WSC	396	214	134	0.990±0.00000	5.917±2.831	0.0128±0.0068	−2.119	0.000	−24.594	0.0020	−3.612	<0.02	−3.423	<0.02

Abbreviations: N, number of sequences; h, number of haplotypes; S, number of polymorphic sites; MNPD, mean number of pairwise differences; SD, standard deviation; P, probability value.

*is a part of the parameter name.

397 samples were successfully assigned to mtDNA haplogroups using a combination of HVS-I sequence motifs and single nucleotide polymorphisms (SNPs) distributed around the coding region of the mtDNA genome. A total of 79 haplogroups or paragroups (unclassified lineages within a clade marked with an asterisk [*]) were identified (Figure 2b, Table S1 and Table S2), all within the two principal out-Africa macrohaplogroups: M and N (including R). Macrohaplogroup M and its subhaplogroups comprise 59.70% of the Qiangic maternal gene pool, and macrohaplogroup N and its subhaplogroups comprise the left 49.30%. The most prevalent haplogroups within macrohaplogroup M, haplogroup D and G represent 18.14% and 13.60% of all the samples. Within macrohaplogroup N, haplogroup A and F are the most common lineages, accounting for 13.60% and 10.58% of Qiangic, respectively. The majority of the mtDNA lineages belong to eastern Eurasian specific groups, including those from Northeast Asia (A, D4, D5, G, C, and Z) [83]–[85] and Southern China or Southeast Asia (B, F, M7, and R9) [54]. Only two U samples in Yajiang might be traced for their origins to western or southern Eurasia, comprising 0.5% of Qiangic. The frequencies of Southern China or Southeast Asia specific haplogroups in Horpa-Danba, Horpa-Daofu, Tibetan-Xinlong, and Tibetan-Yajiang are 26.09%, 22.50%, 27.73%, and 21.35%, respectively. However, Tibetan-Yajiang, Horpa-Danba, Horpa-Daofu and, to a lesser extent, Tibetan-Xinlong, display a considerable Northeast Asian proportion of lineages (56.77%, 56.52%, 55.00%, and 43.70%, respectively). Consistent with other studied Tibetan populations on the Tibetan Plateau, Qiangic populations also showed a strong similarity with Northeast Asian populations.

We performed a PCA using the mtDNA haplogroup frequencies of Qiangic groups in this study and other 68 populations to see the detailed genetic patterns of those populations (Figure 3b, Table S3). The first PC revealed a clear geographic division between northern populations (Altaic and Northern Han) and southern populations (Southern Han, Tai-Kadai, and Hmong-Mien). Qiangic groups were clustered in the northern pole due to the high frequencies of haplogroup A and G. Han Chinese and Tibeto-Burman populations showed significantly different distributions in the second PC. Qiangic populations were clustered within Tibeto-Burman group due to the existence of haplogroup M9a’b and M13.

#### Phylogeography of Macrohaplogroup M

**Figure 5:**
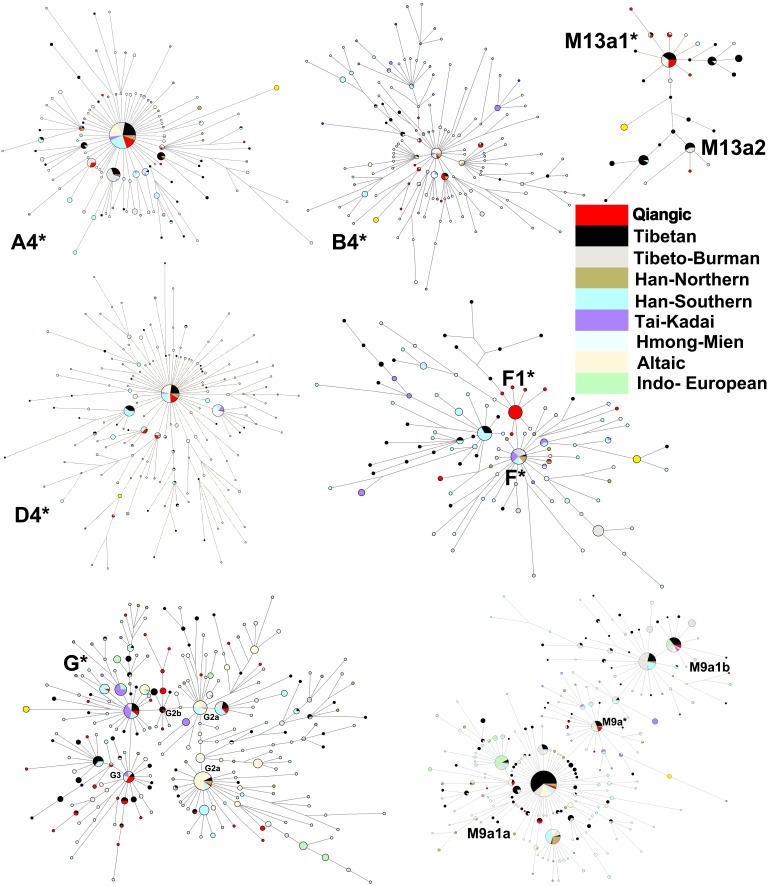
Reduced Median-joining network based on the HVRI data of mtDNA.

Macrohaplogroup M and its subhaplogroups represent the majority of the Qiangic maternal lineages, with frequencies ranging from 65.22% in Horpa-Danba to 57.98% in Tibetan-Xinlong. Haplogroup D4 and G are the most frequent sub-clades of macrohaplogroup M in Qiangic populations, each comprising 13.60%. Haplogroup D4, which is prevalent throughout Central Asia [85], Northeast Asia [86], [87], and Southwest China [5], [8], [65], [66], represents the majority of haplogroup D samples in Horpa-Danba (17.39%), Tibetan-Yajiang (13.54%), Tibetan-Xinlong (13.45%), and Horpa-Daofu (10.00%). The haplotypes of D4* were extensively shared among Qiangic, Tibetan, Han Chinese, and Altaic (Figure 5). Specifically, sub-haplogroup D4j3 was detected in Horpa-Danba and Horpa-Daofu with considerable frequencies (4.35% and 5.00%, respectively). The age estimates generated for D4* and D4j3 in Qiangic were about 15 kya (Table 3). In addition, the population growth factor, Fu’s Fs values of haplogroups D4* and D4j3, were significantly negative (Table 4), implying post-LGM expansions of those two lineages in Qiangic.

**Table 3:** Estimates of the coalescence time of selected mtDNA haplogroups inferred from the ρ statistic within Qiangic populations.

Haplogroup	A4*	B4*	C*	D4*	D4j3	D5*	D5a2a	F1*	F1a	G*	G2a
Time(years BP ± SD)	8510±3647	27220±11372	41728±14806	14722±5167	16152±6594	12563±9932	20558±7661	5139±2098	45228±16855	18845±7123	34263±11234
Haplogroup	G2b1b	G3*	M7b	M9a	M9a1a	M9a1b1	M10	M13a1b	N9a	R*	Z
Time(years BP ± SD)	20558±8392	21985±7328	28267±9422	7067±4080	13302±6651	12563±6282	12563±7693	5653±4214	30152±11919	58248±15608	12563±7693

Abbreviations: BP, before present; SD, standard deviation.

*is a part of the haplogroup name.

**Table 4:** Growth summary statistics and frequency spectrum tests for deviation from neutrality.

Haplogroup	Tajima’s D	Fu’s Fs	Fu and Li’s D	Fu and Li’s D*	Fu and Li’s F	Fu and Li’s F*
A*	−1.118	−0.491	−0.200	−0.814	−0.578	−1.020
A4	−**2.184** a	−**6.181** a	−1.304	−**3.020** b	−1.927	−**3.237** b
B4	−1.431	−**6.724** a	−**2.030** c	−**2.165** c	−**2.124** c	−**2.264** c
B5b	−0.065	−1.793	0.479	0.081	0.420	0.054
C*	−0.229	0.146	0.679	−0.125	0.572	−0.176
D4	−**2.079** b	−**5.692** a	−**3.457** b	−**3.423** b	−**3.576** b	−**3.519** b
D4j3	−**1.576** c	−**3.082** b	−1.619	−1.651	−1.899	−1.812
D5	−1.035	−1.262	−1.042	−1.193	−1.083	−1.264
D5a2a	−1.462	−1.776	−1.498	−1.581	−1.773	−1.762
F1	−**2.324** a	−**3.409** b	−**3.430** b	−**3.637** b	−**3.593** b	−**3.781** b
F1a	−0.599	−1.964	−0.078	−0.598	0.000	−0.649
G*	−**1.809** b	−1.637	−**2.253** b	−**1.940** b	−**2.478** b	−**2.119** b
G2a	−0.915	−**3.027** b	−0.748	−1.275	−0.771	−1.344
G2b1b	−1.270	−1.583	−0.995	−1.581	−1.161	−1.705
G3	−1.121	−**4.928** a	0.926	−0.405	0.529	−0.707
M7b	−0.590	−0.138	−0.246	−0.525	−0.426	−0.592
M9a	−1.541	−0.911	−0.888	−1.596	−1.194	−1.766
M9a1a	−**1.680** c	−1.588	−1.672	−**2.492** b	−1.767	−**2.614** b
M9a1b1	−1.043	0.627	−0.207	−0.971	−0.516	−1.081
M10	−0.963	−0.943	0.320	−0.871	0.190	−0.975
M13a1b	−**1.766** b	0.505	−0.410	−**1.942** c	−0.555	−**2.145** c
N9a	−0.886	−**2.774** c	−0.419	−0.851	−0.526	−0.927
R*	−1.534	−**7.523** a	−**2.170** c	−1.787	−**2.371** c	−1.960

a means P<0.01,

b means P<0.05,

c means 0.05<P<0.1. A haplotype L3e is used as an out group when calculating Fu and Li’s D and F.

*is a part of the parameter name or haplogroup name.

Haplogroup G is found at high frequencies in northeastern Siberia but it is also common among populations of Japanese Archipelago and Korean Peninsula. This haplogroup also comprises an average of 20% of the maternal gene pool of the Tharus from Nepal [88] and accounts for more than 10% in the Tibetan populations of Nagqu, Chamdo, Lhasa, Garze, and Monba [5]. In this study, haplogroup G and subhaplogroups G2a, G2b1b, G3, and G3a1 account for 20% of Horpa-Daofu and reach frequencies greater than 10% in three other Qiangic populations. Subhaplogroup G2a is represented as four distinct HVS-I motif types: 16129–16223–16278–16362 (I), frequent in Tibetan and Southern Han but nearly absent in Altaics; 16223–16227–16278–16362 (II), frequent in all the above three populations and probably experienced population expansion in Altaics (Figure 5); 16193–16223–16278–16362 (III), exclusive in South Asia. All of the G2a samples in Horpa-Daofu harbor haplotype II but add one more mutation at site 16304. However, most of Tibetan-Xinlong samples belong to haplotype I (50%). Subhaplogroup G2b1b was first reported as a novel haplogroup in northeast India and has low frequency distribution in Tibet and surrounding regions [89], [90]. This haplogroup accounts for 4.69%, 2.50%, and 0.84 of Tibetan-Yajiang, Horpa-Daofu, and Tibetan-Xinlong. Compared with other Tibetan samples, 72.73% of Qiangic G2b1b samples were detected with a mutation at site 16356, thus forming some exclusive clades in the network (Figure 5). Subhaplogroup G3 comprises 6.77%, 5.00%, 3.36%, and 2.17% of Tibetan-Yajiang, Horpa-Daofu, Tibetan-Xinlong, and Horpa-Danba, respectively. Two Yajiang samples are further defined as G3a1 by a mutation at site 16215. In addition, we have found two Horpa-Danba G2a samples bearing both G2a (16278) and G3 (16274) characteristic mutations and thus we could not tell the exact haplogroup classification of those two samples. The coalescence time estimates of G*, G2b1b, and G3 were all around 20 kya and the age of G2a even reached about 34 kya (Table 3). However, it is noteworthy that the arrival time of these haplogroups at the Tibetan Plateau might be somewhat more recent than their coalescent ages would indicate, because nearly all these haplogroups (except G2b1b) had already differentiated before their arrival on the plateau (Figure 5). The exclusive clades in the network (Figure 5) and the significant negative Fu’s Fs values (Table 4) of G2a and G3 suggest the probable isolation and secondary population expansion of the two lineages.

Haplogroup M8 has two sublineages, haplogroup C and Z. Haplogroup C is a common lineage, which is widespread in East Asia and Siberia and is one of the founder lineages among Native Americans [6]. Haplogroup C comprises 8–10% of Horpa-Danba and Tibetan-Yajiang, but was detected at a very low frequency or even absent in Tibetan-Xinlong and Horpa-Daofu. Almost 60% of the C samples in present study harbored a specific HVS-I motif 16093–16298–16327 and were assigned as C4d. One Horpa-Danba individual with HVS-I motif 16298–16327 is also classified as C4d through complete sequencing (Doc S2). Haplogroup C4d has been supposed to be Tibetan specific, frequencies ranging from 1.6% to 5.0% in populations of Tibet [5]. However, the frequency of C4d in Tibetan-Yajiang even reaches 6.25%. In addition, all the reported C4d samples in Tibet and Qinghai have the same motif as above mentioned. However, 25% of the C4d samples in Yajiang share another mutation at site 16111. About 23% of C samples in Qiangic with a mutation at site 16357 might be assigned as C4a2′3′4, which is also restricted to Tibeto-Burman populations. Haplogroup Z is observed at relatively low frequencies in Qiangic populations.

M9a’b is widely distributed in mainland East Asia [89] and Japan, and reaches its greatest frequency and diversity in Tibet [5], [8] and its surrounding regions, including Nepal [88] and northeast India [90], [91]. It has been proposed recently that haplogroup M9’b had most likely originated in southern China and/or mainland Southeast Asia. After the LGM, M9a’b might be involved in some northward migrations in mainland East Asia [60]. In the present study, the frequencies of M9a’b in Horpa-Danba, Horpa-Daofu, Tibetan-Xinlong, and Tibetan-Yajiang are 4.35%, 10%, 13.45%, and 6.77%, respectively. Most M9a* samples (62.5%) of Qiangic shared the main haplotype that clustered in the central largest clade with other Tibeto-Burman populations in the network. However, the estimated age of M9a* is relatively young at about 7 kya. M9b is largely restricted to the non-Tibetans in southern China and southwest China [60]. We have detected low frequencies of M9b in Horpa-Danba and Tibetan-Xinlong (2.17% and 0.84%, respectively). In the networks of M9a1a and M9a1b, most of the Qiangic samples shared the descent types, giving a clear signal of out of Tibet migrations of those haplogroups. The age estimates generated for M9a1a and M9a1b1 in Qiangic were around 12–13 kya (Table 3), consistent with proposed post-glacial dispersal of the M9a’b lineages.

Haplogroup M13a has been found at its greatest frequency and diversity in Tibet, but it has also been detected at very low frequencies in Siberian Buryat, Yakut, Altaian Kazakh, and Ewenki [85], and central Asian Kirghizs [92] as well as Barghuts [84], [93], [94]. The frequency of haplogroup M13a in Qiangic populations is remarkable, accounting for 3.27% of all samples. In the network of haplogroup M13a1 and M13a2, Qiangic and Tibetan-Burman samples formed some almost exclusive clades. This strongly suggests that these specific lineages have de novo origins within Tibetans. Specially, 70% of subhaplogroup M13a1b samples in Qiangic share the same haplotype. A coalescence time estimate for M13a1b corresponded to 5.7 kya (Table 3), suggesting a relatively recent Neolithic expansion out of Tibet and even more recent arrival into northern Asia of this lineage.

Qiangic populations also exhibit some basal Eurasian mtDNA lineages. Haplogroup M62, for example, was first reported in Northeast India [90] and since then has been reported in several populations at low frequency throughout Tibet [5], [8]. Zhao et al. suggested that M62 might represent the genetic relics of the initial Late Paleolithic settlers (>21 kya) on the Tibetan Plateau. In this study, we observed haplogroup M62b in three Yajiang Tibetans. The haplotype of those three individuals is different from all other reported M62 samples with a mutation at site 16305. Likewise, haplogroup M74a was detected in one Xinlong Tibetan, and the haplotype of which bearing a distinctive mutation at site 16274 only shared with one Maonan individual, one Zhuang individual, and one Hainan Han Chinese [52]. Haplogroup M33c was found in a Tibetan sample from Yajiang with a similar haplotype as some Hmong-Mien samples [52].

#### Phylogeography of Macrohaplogroup N

Haplogroup R and its subhaplogroups (B and F) represent the majority of the lineages branching from the basal N trunk, accounting for 26.09%, 22.50%, 28.57%, and 23.44% of the maternal diversity in Horpa-Danba, Horpa-Daofu, Tibetan-Xinlong, and Tibetan-Yajiang, respectively. Subhaplogroup B4* is the most frequent lineage of haplogroup B in Qiangic, comprising 4.53% of all the samples. In the network of B4*, the root clade composed almost exclusively of non-Tibetan-Burman samples, however, the Tibetan-Burman samples only formed some small clusters or shared the terminal types, suggesting that B4* had already differentiated before its arrival in Tibet. Subhaplogroup F1* is the most frequent lineage of haplogroup F in Qiangic, accounting for 5.54% of all the samples, and even comprising as high as 12.5% of Horpa-Daofu. Age estimate generated for F1* in Qiangic was around 5 kya (Table 3). The exclusive Qiangic cluster of F1* in the network suggests a strong bottleneck or founder effect in its Neolithic migration towards the plateau. The significant negative values of the growth factor estimates (Table 4) suggest a secondary expansion and probable selection of F1* lineage during its adaptation in the plateau.

Haplogroup N* is almost exclusively represented by haplogroup A in our samples. Haplogroup A is widely distributed in northern and eastern Asia, occurring at frequencies of 5%–10% in different populations [85]. Haplogroup A also has an average frequency of nearly 9% on the plateau [5]. Subhaplogroup A4*, which is mainly found in Central, Northeast and Southwest Asia, is the most frequent sublineage of haplogroup A in Qiangic, accounting for 2.17%, 5.00%, 4.20%, and 12.50% of Horpa-Danba, Horpa-Daofu, Tibetan-Xinlong, and Tibetan-Yajiang, respectively. Network analysis of haplogroup A4* revealed a star-like pattern and thus showed a signal of population expansion on the plateau (Figure 5). The probable population expansion was also confirmed by growth summary statistics in this lineage (Table 4). Subhaplogroup A11 split from the root of haplogroup A very early and formed a distinct lineage. A11a and A11b, the two sublineages of A11, have the different distribution pattern. Most of the A11 samples in Tibet belong to A11* or A11a and only a few have a control-region substitution at site 16234, assigned as A11b. However, almost all the A11 samples in the Tibetan-Burman and Han Chinese of Yunnan belong to A11b. In the present study, three of five A11 samples belonged to A11* and the other two were assigned as A11b.

## Discussion

The Sino-Tibetan linguistic family comprises some 460 languages distributed in East Asia, Southeast Asia, and parts of South Asia, including the Chinese and Tibeto-Burman subfamilies [1]. Despite intense linguistic, archaeological, and genetic researches, where the Sino-Tibetan speakers came from, how they dispersed remain major open questions. One widely accepted hypothesis states that the ancestors of the Sino-Tibetan population were originally from the Neolithic Age Di-Qiang people in the upper and middle Yellow River basin. Di people have gradually developed into Han Chinese and Qiangic populations since the collapse of Later Liang dynasty (one of the Sixteen Kingdoms dynasty, AD 386–403). Here, we integrated the Y chromosome and mtDNA evidence of Qiangic populations to provide a broader framework for reconstructing the history of Sino-Tibetan.

From the paternal Y chromosome perspective, haplogroup D1-M15 originated from D*-M174 during its migration into mainland East Asia [95]. Around 50–60 kya, a subgroup of haplogroup D*-M174 and D1-M15 started their northward migration through WSC corridor into nowadays Qinghai province, and then probably moved along the well-known route, called the Tibeto-Burman corridor, to enter the Himalayas [95]. Haplogroup D*-M174 probably gave birth to D3a-P47 in Tibet [95]. Haplogroup D3a-P47 experienced recent population expansion on the Tibetan Plateau, and then probably migrated southward via the WSC corridor and gradually became the main genetic component of Tibeto-Burman populations in nowadays Sichuan, Yunnan, and Guangxi province. Y chromosome haplogroup D might give the evidences of the late Palaeolithic human activity on the plateau. The genetic relics of late Palaeolithic age have also been detected in the maternal side, for example, haplogroup M62b. In addition, a number of Paleolithic sites have been excavated crossing the Tibetan Plateau [96]–[99], documenting the earliest human presence on the plateau dated to 20–30 kya.

Around 20–40 kya, a population with dominant haplogroup O3-M122 Y chromosomes (haplogroup O3a1c-002611, O3a2c1*-M134, O3a2c1a-M117, and probably other O3 lineages) finally reached the upper and middle Yellow River basin and formed the Di-Qiang populations. During the Neolithic period, the Di-Qiang people experienced relatively huge population expansion. A subgroup of the Di-Qiang people with dominant haplogroup O3a2c1*-M134 and O3a2c1a-M117, now called the Proto-Tibeto-Burman people left their Yellow River homeland, probably also moved along the Tibeto-Burman corridor, embarking on large-scale westward migrations to nowadays Qinghai province and then southward to the Himalayas, or southward migration directly via the WSC corridor to Yunnan and Guangxi, where they mixed with D-M174 linages and developed into Tibeto-Burman populations. However, haplogroup O3a2c1*-M134 might have already reached Tibet predated the above southward migration together with O3a2c1a-M117, judging from the high diversity in the network of O3a2c1*-M134 (Figure 4). In addition, another branch of the Di-Qiang people, the proto-Chinese, with dominant haplogroup O3a1c-002611 migrated eastward to the central China plain area, the middle and lower Yellow River Valley, and integrated gradually with the natives (probably populations with haplogroup C-M130 or D-M174) around 5–6 kya. Subsequently, the Di-Qiang people that resided in upper and middle Yellow River basin with haplogroup O3a2c1*-M134 and O3a2c1a-M117 formed the well-known Yan-Huang tribe (Hot Emperor and Yellow Emperor), and the eastward branch with O3a1c-002611 developed into the Dong Yi tribe. The Yan-Huang tribe together with the Dong Yi tribe gradually developed into a large population known as Han Chinese. With the expansion of Han Chinese, especially southward, this group became the largest one of the 56 officially recognized ethnic populations in China.

The role of haplogroup O3-M122 lineages played in the origin of Tibeto-Burman populations has suggested extensive genetic input from northern Asians. This suggestion has been supported by previous studies employing autosomal STR [100], [101], Y chromosome [33], [34], and mtDNA [5]–[9]. It is not surprising that the maternal variation of Qiangic populations was also largely contributed by northern Asian-prevalent haplogroups, including haplogroups A, C, D, and G. In addition, cultural features of the upper Yellow River basin, such as painted pottery, millet agriculture, and urn burial, are prevalent in the Neolithic sites of WSC, probably due to the demic diffusion via the genetic corridor [102]. However, we still could not rule out the possibility that the complex genetic structure of Qiangic populations might be due to repeated admixture from surrounding populations, which provides directions for future work.

## Supporting Information

Table S1 **Y chromosome and mtDNA haplogroup frequencies of Qiangic populations.**
(XLS)

Table S2 **Y chromosome SNP and STR data, mtDNA haplogroups and HVS-I motif of Qiangic** **populations.**
(XLS)

Table S3 **Y chromosome and mtDNA haplogroup frequencies used in PCA plot and haplogroup** **contributions to each PC.**
(XLS)

Table S4 **Shared Y-STR haplotypes between Qiangic populations and other East Asian populations.**
(XLS)

Doc S1 **Geographic distribution of Y chromosome haplogroup D1 and D3a, Y-STR neighbor-joining tree based on genetic distance.**
(DOC)

Doc S2 **Three representative complete mtDNA haplotypes compared to rCRS.**
(DOC)
