# Supplementary material for: Genetic Structure of Qiangic Populations Residing in the Western Sichuan Corridor
Source: PLoS One. 2014 Aug 4;9(8):e103772. doi: 10.1371/journal.pone.0103772 (PMC4121179; doi:10.1371/journal.pone.0103772)
Supplement: Doc S1 — Geographic distribution of Y chromosome haplogroup D1 and D3a, Y-STR neighbor-joining tree based on genetic distance. (DOC) [file pone.0103772.s005.doc]

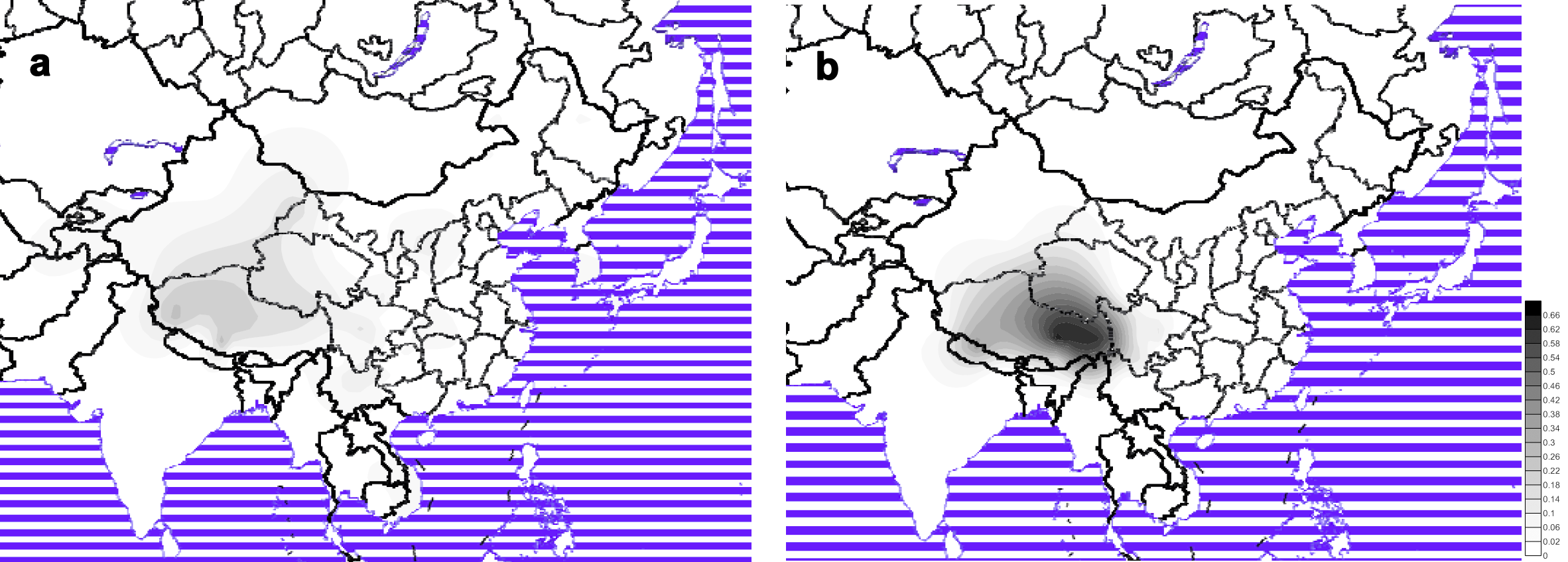


Figure S1. Geographic distribution of Y chromosome haplogroup D1 and D3a. a. Geographic distribution of Y chromosome haplogroup D1 in East Asia. b. Geographic distribution of Y chromosome haplogroup D3a in East Asia.


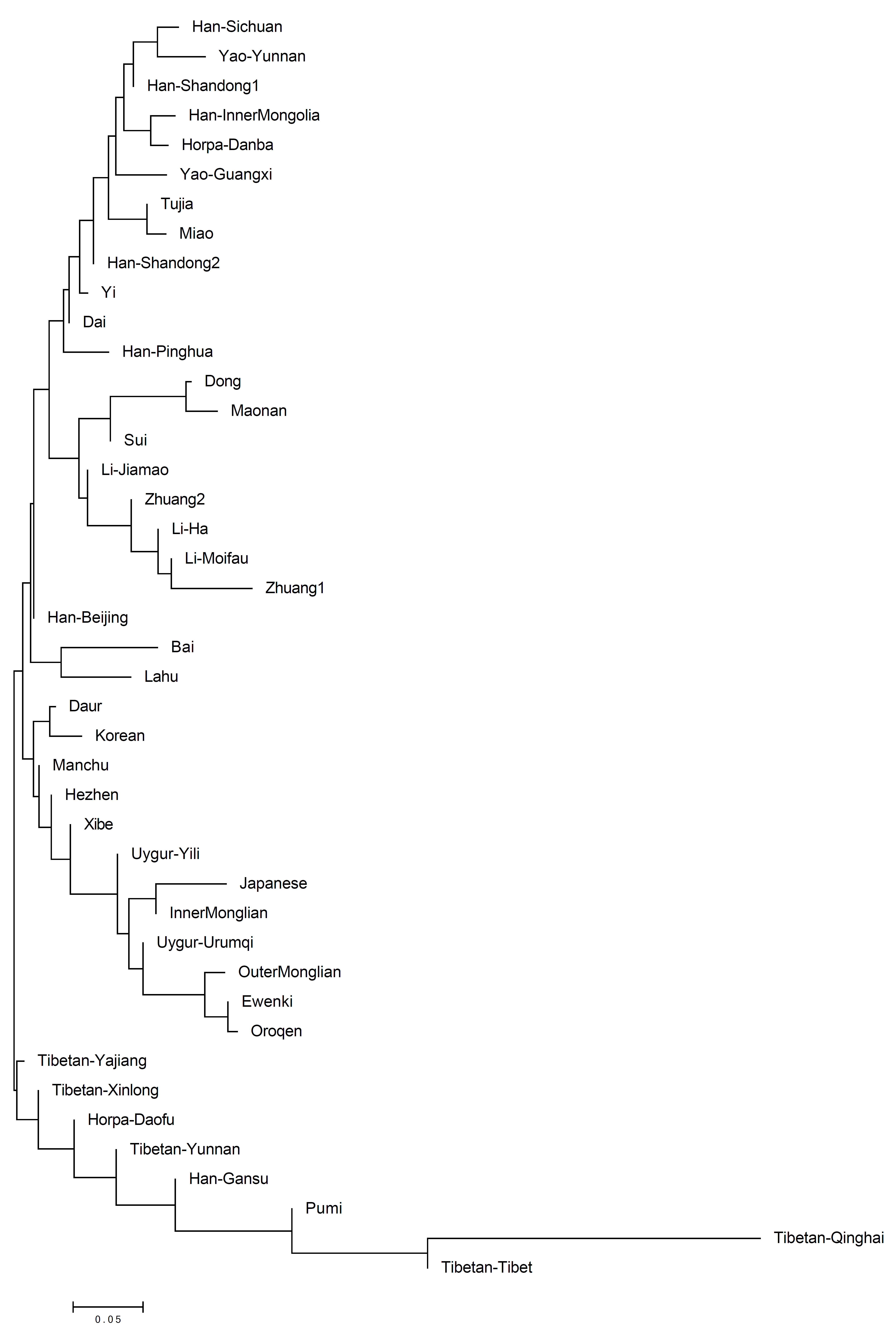


Figure S2. Y-STR neighbor-joining tree of 43 populations. Arlequin 3.11 was used to calculate the Y-STRs (DYS19, DYS389I, DYS390, DYS391, DYS392, and DYS393) R_st_ genetic distances. The neighbor-joining tree based on R_st_ distances was constructed in Mega 5.2.1.
