# Supplementary material for: Genetic Structure of Qiangic Populations Residing in the Western Sichuan Corridor
Source: PLoS One. 2014 Aug 4;9(8):e103772. doi: 10.1371/journal.pone.0103772 (PMC4121179; doi:10.1371/journal.pone.0103772)
Supplement: Doc S2 — Three representative complete mtDNA haplotypes compared to rCRS. (DOC) [file pone.0103772.s006.doc]

| **Sample** | **Population** | **Variant rCRS** | **Haplogroup** |
| --- | --- | --- | --- |
| **BM024** | Horpa, Bamei | A73G, A193G, G709A, A750G, T789C, A1438G, A2706G, 3106d, A4769G, T5465C, A5498G, A8860G, C7028T, G9123A, G11719A, A13834G, C14751T, C14766T, T15262C, 16189, T16217C, A16299G, T16519C | B4a4 |
| **DBB005** | Horpa, Danba | A73G, T152C, 249d, A263G, T489C, C628T, A750G, A1438G, A2706G, 3106d, T3552A, A4715G, A4769G, A6002G, G6026A, C7028T, A7100G, C7196A, G8584A, A8701G, A8860G, T9540C, A9545G, A10398G, C10400T, T10873C, G11719A, G11914A, G11969A, C12705T, A12780G, A13263G, G13708A, T14318C, C14766T, T14783C, G15043A, T15204C, A15236G, G15301A, A15326G, T15458C, A15487T, T16298C, C16327T, T16519C | C4d |
| **DBB006** | Horpa, Danba | A73G, A263G, C548T, A750G, A1438G, A2706G, 3106d, C4086T, A4769G, T6392C, G6962A, C7028T, A7804G, A8860G, G9053A, G9548A, G9554A, C10211T, G10310A, T10609C, G11719A, G12406A, C12882T, A13149G, G13759A, G13928C, C14766T, A15326G, A16162G, T16172C, T16189C, T16304C, T16519C | F1a1c |
